# Effects of Different Percentages of Microhydroxyapatite on Microhardness of Resin-modified Glass-ionomer and Zirconomer

**DOI:** 10.4317/jced.53668

**Published:** 2017-06-01

**Authors:** Farahnaz Sharafeddin, Soodabe Shoale, Mahsa Kowkabi

**Affiliations:** 1 Professor of Dept. of Operative Dentistry, Biomaterial Research Center, School of Dentistry, Shiraz University of Medical Sciences, Shiraz, Iran; 2 Postgraduate Student, Dept. of Operative Dentistry, School of Dentistry, Shiraz University of Medical Sciences, Shiraz, Iran

## Abstract

**Background:**

Hydroxyapatite (HA) is the main mineral component of the tooth structure, which exhibits considerable biological behavior and its incorporation might improve microhardness of dental materials. Microhardness of restorative materials, like glass-ionomer, is critical for the clinical longevity of restorations. The aim of this study was to evaluate the microhardness of two glass-ionomers types by incorporating different percentages of microhydroxyapatite.

**Material and Methods:**

In this study, 80 disc-shaped experimental specimens (6 mm in diameter, 2 mm in height) were prepared in 8 groups, including resin-modified glass-ionomer (RMGI, GC, Gold Label, Japan), zirconia-reinforced glass-ionomer (Zirconomer, Shofu, Kyoto, Japan), and their mixture with 0, 5, 15 and 25 wt% of microhydroxyapatite (Sigma-Aldrich, Germany). All the specimens were stored in deionized water at 37ºC for 24 hours. Then Vickers microhardness test was carried out on the both sides of specimens and data were analyzed using two-way ANOVA and paired t-test (*P*<0.05).

**Results:**

Microhardness of Zirconomer and RMGI increased significantly due to adding 5 and 15 wt% of micrhydrox-yapatite (*P*<0.001). The highest Vickers hardness number (VHN) was recorded in the RMGI group with 5 wt% of microhydroxyapatite. In addition, in all the study groups RMGI exhibited higher microhardness values than Zirconomer (*P*<0.001). However, microhardness values decreased significantly after adding 25 wt% of microhydroxyapatite to Zirconomer (*P*<0.001). Similarly, VHN decreased in RMGI groups containing 25 wt% of HA compared to control groups (without HA) (*P*<0.001).

**Conclusions:**

Incorporation of 5 and 15 wt% of microhydroxyapatite to RMGI and Zirconomer improved microhardness, while adding 25 wt% of HA decreased hardness with both experimental materials compared to the control groups (without HA).

** Key words:**Microhardness, Resin-modified glass-ionomer, Zirconia-reinforced glass ionomer, Microhydroxyapatite.

## Introduction

Glass-ionomer cements (GICs) are used in clinical dentistry as commercial materials since the early 1970s (1,2). The success of these cements is attributed to their unique properties such as direct bonding to tooth structure, anti-cariogenic action due to release of fluoride and biocompatibility with pulp tissue (3,4). Minimal microleakage as a result of low coefficient of thermal expansion similar to the tooth structure is one of the important advantages of glass-ionomers (5,6). Despite these advantages, the clinical use of glass-ionomers was limited due to its certain demerits such as low mechanical properties, lack of strength and toughness, short working time, low resistance to wear and early susceptibility to moisture contamination (2,7).

Resin-modified glass-ionomer (RMGI) was developed with better mechanical properties compared to conventional glass-ionomer (8,9). Incorporation of various fillers like silver, gold, titanium, palladium, zirconia, stainless steel powder and SiC whiskers into glass-ionomers has been investigated in order to improve their mechanical properties, but poor aesthetic and low abrasion resistance are their important limitations (10,11). Zirconia fillers have often been applied in dental procedures like implants due to their chemical and good mechanical strength and toughness (1,12).

Zirconia is one of the tooth-colored materials with good dimensional stability and excellent strength and toughness, coupled with a Young’s modulus in the same order of magnitude of stainless steel alloy and is the origin of the interest in using ZrO2 as a filler (1,12,13).

Hydroxyapatite (HA), the main mineral component of the tooth structure and bone, is a bioceramic containing calcium and phosphorus (9,14). The HA particles were added to glass-ionomer powder due to their biocompatibility and similar composition to apatite in human dental and skeletal systems (15). Several studies reported improvements in mechanical properties of these materials such as diameter, tensile strength, fracture toughness, bonding and compressive strength compared to conventional glass-ionomers (8,14). ZrO2 accompanied by HA has been used for strengthening in biomedical applications. Zirconia has higher strength than GIC and HA particles and does not dissolve in distilled water (1).

The bioactive glass is a type of glass containing HA crystallized with thermal treatment and has the chemical composition NaO-CaO-SiO, containing some P2O5 (15,16). Also, bioactive glasses and ceramics can interact with bone and dentin in biologi-cal environments like saliva (17). Some studies exhibited the remineralizing effect of bioactive glass materials on dentin and antimicrobial properties (18,19).

Since microhardness is one of the important mechanical properties of material that ensures resistance of plastic modifications, improvement of this character affects the success of clinical application of restorative materials (20). In spite of the fact that adding of HA improves microhardness of GICs, increasing it to more than the specified amount of HA may reverse this effect. The effective amount of HA has not been determined. Therefore, in the present study, effects of different percentages of microhydroxyapatite on microhardness of Zirconomer and RMGI were investigated.

## Material and Methods

Disc-shaped specimens were prepared in cylindrical plastic molds (6 mm in diameter, 2 mm in height) (20). Experimental materials in this study are shown in  Table 1.

**Table 1:**
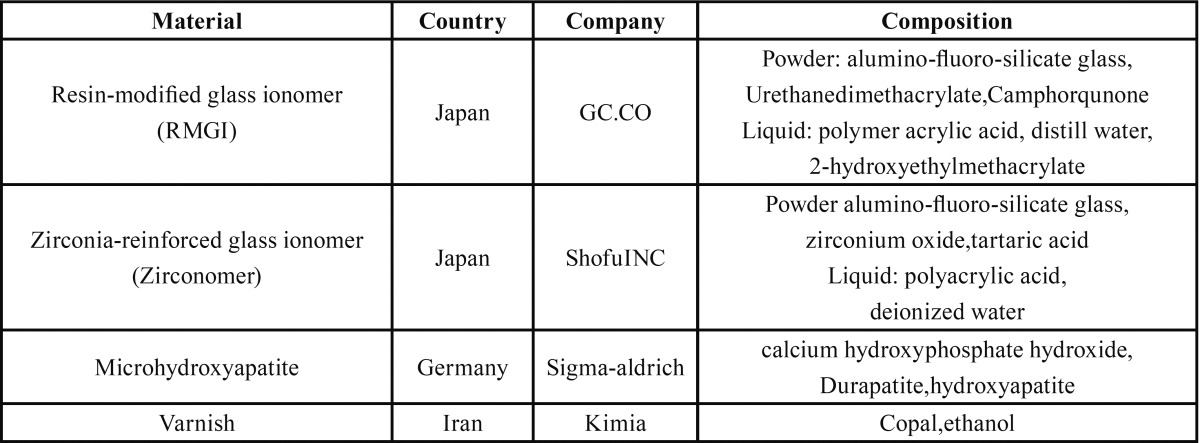
All the materials used in the study.

In group 1, each specimen contained Zirconomer powder as a control group that was mixed with liquid on a mixing pad with a plastic spatula according to manufacturer’s instructions (powder-to-liquid ratio: 8.0:1.0 g) for 30 seconds. Each plastic mold was placed on a Mylar strip and glass slab. Then the mold was overfilled with experimental mixture and another strip and slab were placed on the top surface of the mold for compressing until the materials were completely set (21).

In groups 2, 3 and 4, the specimens contained Zirconomer with 5, 15 and 25 wt% of microhydroxyapatite, respectively.

In group 5, the discs were prepared from RMGI powder that was mixed with liquid according to manufacturer’s proportional recommendations (powder-to-liquid ratio: 3.2 gr/1 gr). Then the molds were overfilled as discussed above.

Both sides of each sample were light-cured for 20 seconds to ensure a perfect setting by using an emitting diode (LED) polymeri-zing unit (Monitex, Bluelex, GT 1200, Taiwan) at a light intensity of 1200 mw/cm2 and a wavelength of 470 nm according to manufacturer’s directions. The curing tip with a diameter of 8 mm was attached on each side of sample discs (21). In groups 6, 7 and 8, the specimens contained RMGI with 5, 15 and 25 wt% of microhydroxyapatite, respectively. Then the procedure was carried out similar to those in group 6. Following the removal of mylar strips, both sides of all the 80 specimens were coated with varnish (Kimia, Iran). Then all the specimens were stored in distilled water at 37ºC for 24 hours in an incubator (ES 250 Nuve, Turkey), individually for each group. Subsequently, both sides of each specimen were polished with the use of a low-speed hand-piece with polishing paper discs (Super Snap, Rainbow Technique Kit, Shofu, Japan) in 4 different grits. Finally, the specimens were washed under running distilled water for 1 minute to remove any debris, followed by testing procedures.

-Microhardness test

**Figure 1:**
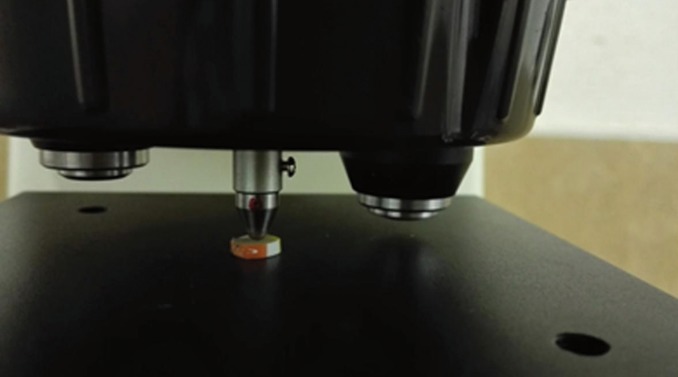
The experimental disc under microindentation of digital Vickers microhardness tester.

**Figure 2:**
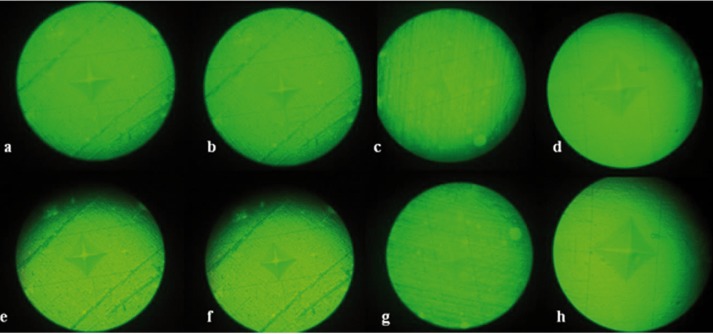
The indentation surfaces in all groups a: RMGI. b: RMGI+5%HA. c: RMGI+15%HA. d: RMGI+25%HA. e: Ziconomer. f: Zirconomer+5%HA. g: Zirconomer+15%HA. h: Zirconomer+25%HA.

The microhardness test was carried out with a digital Vickers microhardness tester (SCTMC, 1000Z, China) using a load of 300 gr with a dwell time of 15 seconds. To measure Vickers hardness number (VHN), three Vickers tests were carried out on each surface of specimens and the mean value was calculated and determined as VHN (Fig. 1). Distances between indentation points and disc borders were not less than 1 mm. The indentation surfaces can be seen in figure 2.

Data were collected and analyzed with two-way ANOVA, Tukey HSD test and paired t-test , using SPSS 22 (*P*<0.05).

## Results

Two-way ANOVA showed that the interaction between microhydroxyapatite, RMGI and Zirconomer was statistically significant (*P*<0.001). These results indicated that microhardness of Zirconomer and RMGI increased significantly due to incorporation of 5 wt% of microhydroxyapatite (*P*<0.001); in addition, VHN of both experimental materials significantly increased after adding 15 wt% of HA (*P*<0.001). Incorporation of 5 wt% of microhydroxyapatite increased the VHN of two materials significantly compared to the groups without HA (control groups), especially in the RMGI group (*P*<0.001). In contrast, VHN decreased after adding 25 wt% of microhydroxyapatite to both materials compared to the control groups and this reduction was higher than that expected in RMGI group in a way that the microhardness value was approximately equal in both materials with 25% HA (*P*=0.605). The mean values of microhardness in all the groups are summarized in  Table 2. RMGI showed higher microhardness values than Zirconomer in all the test groups (*P*<0.001).

**Table 2:**
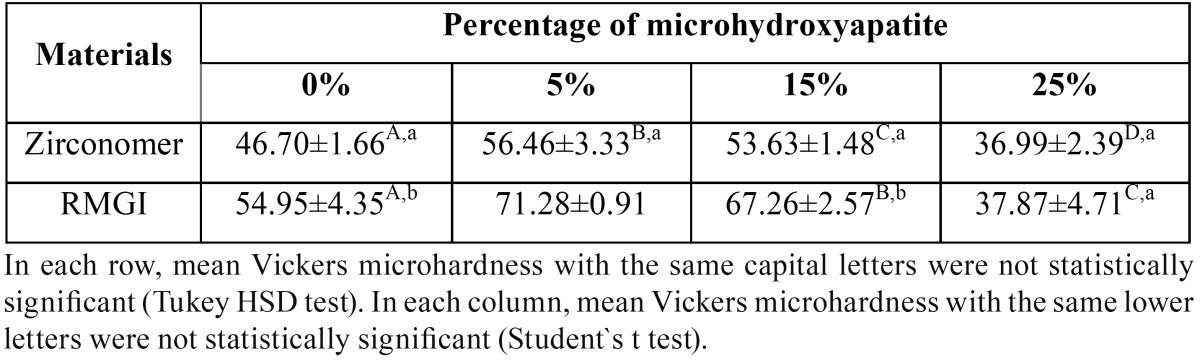
Mean Vickers microhardness numbers (VHN) and Std. deviations in all groups.

**Figure 3:**
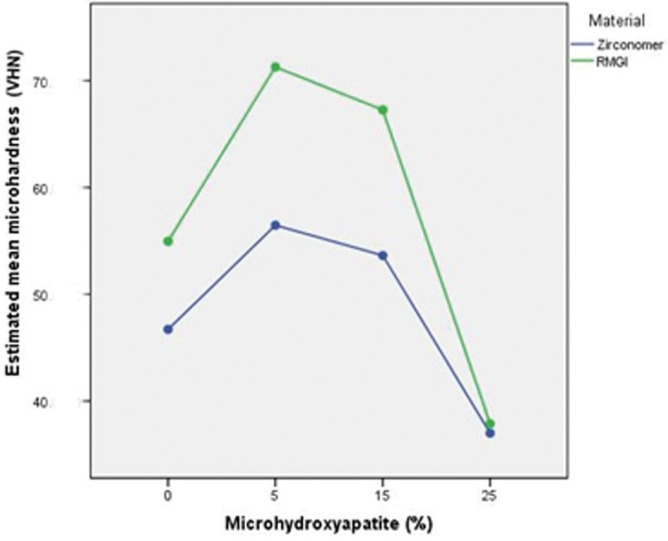
Estimated mean Vickers microhardness values of Zirconomer and RMGI with adding 5%, 15% and 25% microhydroxyapatite.

Comparisons between different percentages of microhydroxyapatite in each experimental material were conducted and analyzed using post hoc Tukey tests (Fig. 3).

Among all the 8 groups, RMGI with 5 wt% of microhydroxyapatite had the highest hardness value. The VHN showed that adding 5 and 15 wt% of HA to RMGI increased microhardness higher than that of Zirconomer, with no statistically significant difference (*P*=0.065). In addition, there was no significant difference between adding 5 and 15 wt% of HA to Zirconomer (*P*=0.047).

The VHN of the top and bottom surfaces of all the specimens are summarized in  Table 3. Pair t-test demonstrated significantly higher VHN for the bottom surfaces compared to the top surfaces (*P*=0.004).

**Table 3:**
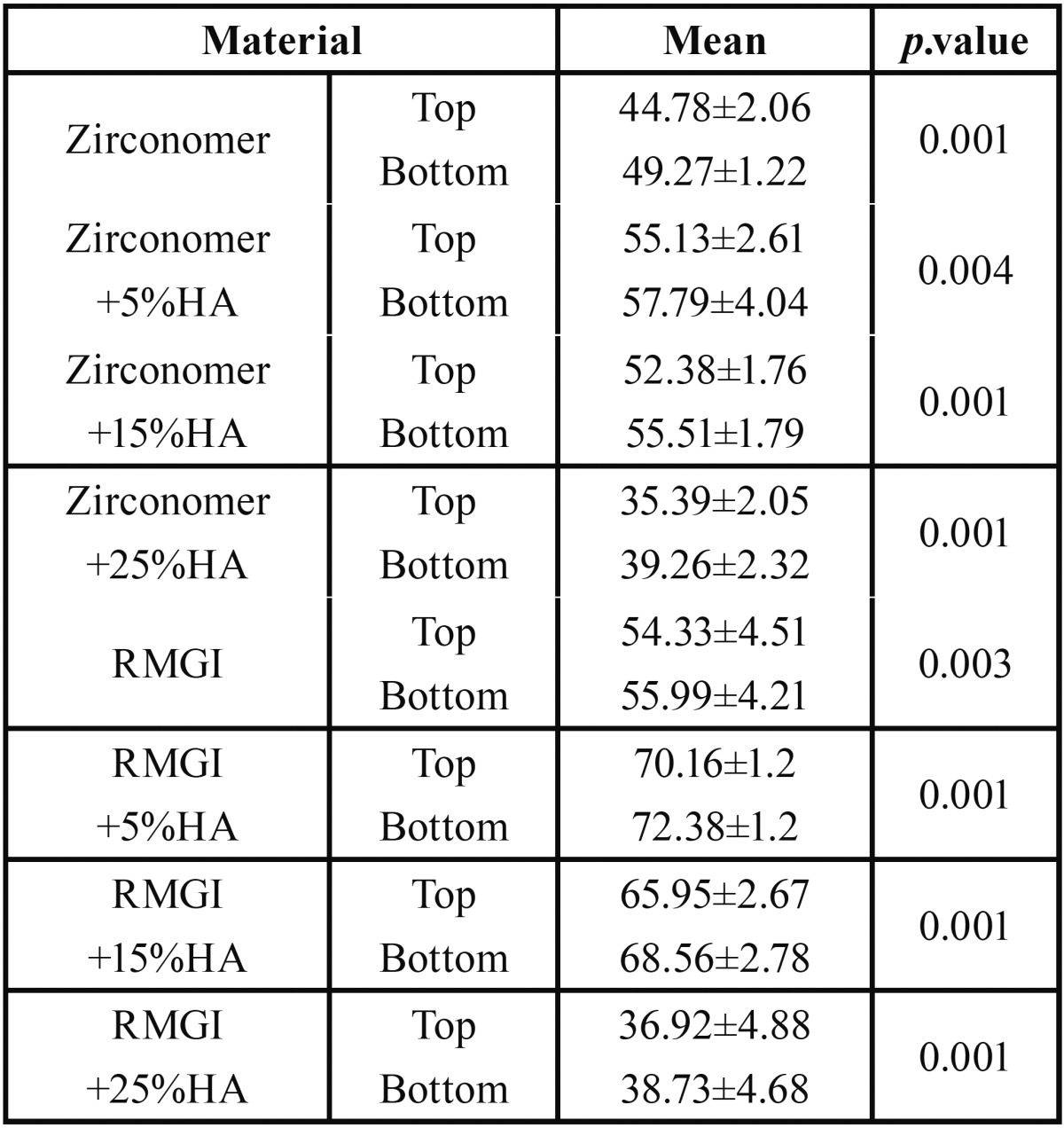
Mean Vickers microhardness numbers (VHN) of the top and bottom surfaces in all the groups(pair t test).

## Discussion

Hardness is one of the physical properties that compromises the fatigue strength of materials due to premature failure (9).The clinical longevity of materials is associated with their resistance to catastrophic failure that is measured using their fracture toughness (9). Incorporation of some fillers such as zirconia or HA has improved fracture resistance, strength and hardness of GICs (4,11). HA is the main mineral component of tooth structure; therefore, incorporation of HA into glass-ionomer can affect some of its properties, including its fracture resistance (4).

The incorporation of HA can result in strengthened matrix of GICs and subsequently, better bonding between the glass core and glass matrix. The fluoride ion release was slightly higher in HA-added glass cement (4,10,13). The apatite formation of HA in combination with the release of ions from glass-ionomer can improve the mechanical properties of glass cements (20). Therefore, in the present study the effect of HA on hardness, as one of the most important mechanical properties of the material, was investigated. Micro-HA was chosen in this study because hardness value of HA is similar to that of natural teeth. Micro-particles of HA are easily mixed with resin (either Bis-GMA+TEGDEMA or Bis-GMA+HEMA) and are used in dentistry to reinforce materials (21). Although nano-particles of HA are more similar to the mineral phase of tooth structure than micro-particles as far as crystal size is concerned, nano-HA considerably prolongs the setting time of GICs (21). Additionally, in this study, Zirconomer was selected instead of conventional glass-ionomers because the results of previous studies indicated that the mechanical properties of HA/ZrO2-GICs were better than those of HA-GICs (1). RMGI was another experimental material in the current study because it provided the highest tensile bond strength for both the enamel and dentin and exhibited better esthetic, adhesion and mechanical properties than conventional GICs. Furthermore, RMGI was more stable in an acidic environment (9). Considering the prolonged setting time of GIC after adding HA, the problem of the extended setting time has been overcome using HA-added RMGI (10). Based on previous studies, the mechanical properties of 4 and 12% HA-GICs have increased compared to the initial cements (13). It was also reported that incorporation of a large amount of HA (50 to 60 wt%) into light-cured monomer could increase Young’s modulus and surface hardness and this large amount of HA was used as the only reinforcing filler in that study (22). However, in the present study, smaller volumes of HA (5,15 and 25 wt%) were added to glass powder similar to previous studies (2,3). Geonka *et al.* synthesized the nanocrystalline calcium-deficient HA into GIC in different compositions (5, 10 and 15 wt%) and reported that the group containing 5% HA had higher surface microhardness than the 15% HA-GIC group. They also showed that an increase in HA volume decreased the Vickers microhardness. The results of the present study are consistent with those reported by Geonka et al, who concluded that the decrease in hardness resulted from a decrease in the density of the set cement. In their study, maximum hardness values were obtained in conventional glass-ionomer without HA (14) but in the present study RMGI with 5 wt% of HA exhibited the highest hardness value.

Yli Up *et al.* studied the Vickers microhardness of the combinations of GIC and RMGI with 10% or 30% HA. They reported that the hardness of glass-ionomers decreased as the amount of hydroxyapatite increased; a finding which is consistent the results of the present study. However, they showed that the hardness values of conventional GICs were higher than light-cured glass-ionomers, but the surface hardness of HA-added RMGI increased during water storage (17,23). However, in this the present study, the hardness values of RMGI groups were higher than the Zirconomer groups.

In the present study, it might have been the larger glass particles sizes and less voids and cracks of RMGI that resulted in higher microhardness values. Due to resin cross-linking and rapid setting, it seems that RMGI was more resistance to being dissolved in water after a day of water storage but Zirconomer is self-curing and therefore chemically similar to glass-ionomers, resulting in more dissolution in water.

In 2006, Mohammed *et al.* evaluated the effects of different ratios of HA (10, 15, 20, 25 and 30 wt%) on microhardness of GIC and to the best of the authors’ knowledge, it is the only study that has reported that by increasing the amount of HA, microhardness of conventional glass-ionomer improves and the best hardness is observed in 20% and 25% groups. They explained that the different hardness in comparison to 20% group might be due to differences in powder-to-liquid ratio and powder particle size (20). But in the present study, the highest values of microhardness were obtained in groups with 5% HA and by adding more than 15 wt% of microhydroxyapatite, microhardness decreased in all the groups, with the lowest values being obtained by incorporating 25 wt% of HA in both experimental materials and the decrease was less than that in the control group.

Moshaverinia *et al.* reported that incorporation of HA and fluoroapatite into glass-ionomer cements increased the other mechanical properties such as (compressive, diametral tensile and biaxial flexural strengths) and bond strength to dentin (24). Also in Lee’s survey, it was shown that the physical properties of RMGI improved with the incorporation of 10% nano-HA and micro-HA (10), and HA-added RMGI showed the highest bioactivity and bond strength to tooth structures (9,10). In another study, Khaghani explained that adding 5 wt% of HA increased the compressive strength of conventional glass-ionomer and also improved its diametral and tensile strengths (3). In the present study, microhardness of modified glass cements was evaluated and the results showed that incorporation of 5 and 15 wt% of HA to glass powder increased microhardness values. The results of this and previous studies suggest that HA-added glass is a promising combination among restorative dental materials, which exhibits favorable mechanical properties. Use of tests and clinical trials are recommended for this combination before its clinical application.

A uniform powder in terms of size and structure of particles is obtained by sintering the glass and HA powders. In the present study, efforts were made to produce a uniform mixture by mixing the powders in an amalgamator but it is possible that in some parts of the surface the material was only HA or glass-ionomer. This factor probably contributes to the decrease in the hardness of the experimental materials. It seems that the volume of HA can change the amount of liquid needed to complete the reaction of particles. Since HA/ZrO2 particle size is smaller than glass powder, the surface area is much larger compared to glass; therefore, it might need a greater amount of liquid for interaction (1). However, in the current study, the same ratio as the manufacturer’s instruction was used; therefore, it seems that an inadequate amount of liquid in the mixture can alter the mechanical properties. Another reason for the reduction in surface hardness when HA is added to RMGI and Zirconomer might be the decrease in the density of set cement consisting of HA, which contains calcium ions. These ions seem to react more than aluminum cations to carboxylate groups in polyacrylic acid in a way that creates fewer cross-links between aluminum and carboxylate and weakens the structure (25). The VHN of the top and bottom surfaces of all the specimens were determined in the present study; the hardness values of the bottom surfaces were higher in all the experimental groups. Mobarak *et al.* showed that the bottom surfaces of Fuji IILC (RMGI) had higher hardness value than the top surface. Their findings are consistent with the results of the present study (26). However, in Cefley’s and Bayindir’s studies, top surface hardness in RMGI discs was higher than the bottom because they had cured only the top surfaces of their samples (27,28). However, some previous studies showed no significant differences in VHN of top and bottom surfaces of RMGI (29,30). The differences in VHN between top and bottom surfaces in this study might be attributed to more acid-base reactions because of the bulk of the samples at the bottom and probably to packing force on bottom surfaces.

Incorporation of nano- and micro-particles of HA could enhance the hardness of conventional glass-ionomer (10). Due to high surface area and good mechanical interlocking with the polymer matrix in nano HA, it is recommended that different percentages of nano HA be used in future studies (21).

## Conclusions

Under the limitations of this study, it can be concluded that incorporation of 5 and 15 wt% of microhydroxyapatite into RMGI and Zirconomer improved the surface microhardness but adding more than 15% of HA resulted in adverse effects. In addition, incorporation of 25% microhydroxyapatite into Zirconomer and RMGI decreased hardness values less than the groups without HA.
